# Supplementary material for: Yeast-Hydrolysate-Derived 1-Methyl-1,2,3,4-tetrahydro-β-carboline-3-carboxylic Acid Inhibits Fat Accumulation during Adipocyte Differentiation
Source: Foods. 2023 Sep 18;12(18):3466. doi: 10.3390/foods12183466 (PMC10528377; doi:10.3390/foods12183466)
Supplement: Supplementary file 1 [file foods-12-03466-s001.zip › foods-2610604-supplementary.pdf]

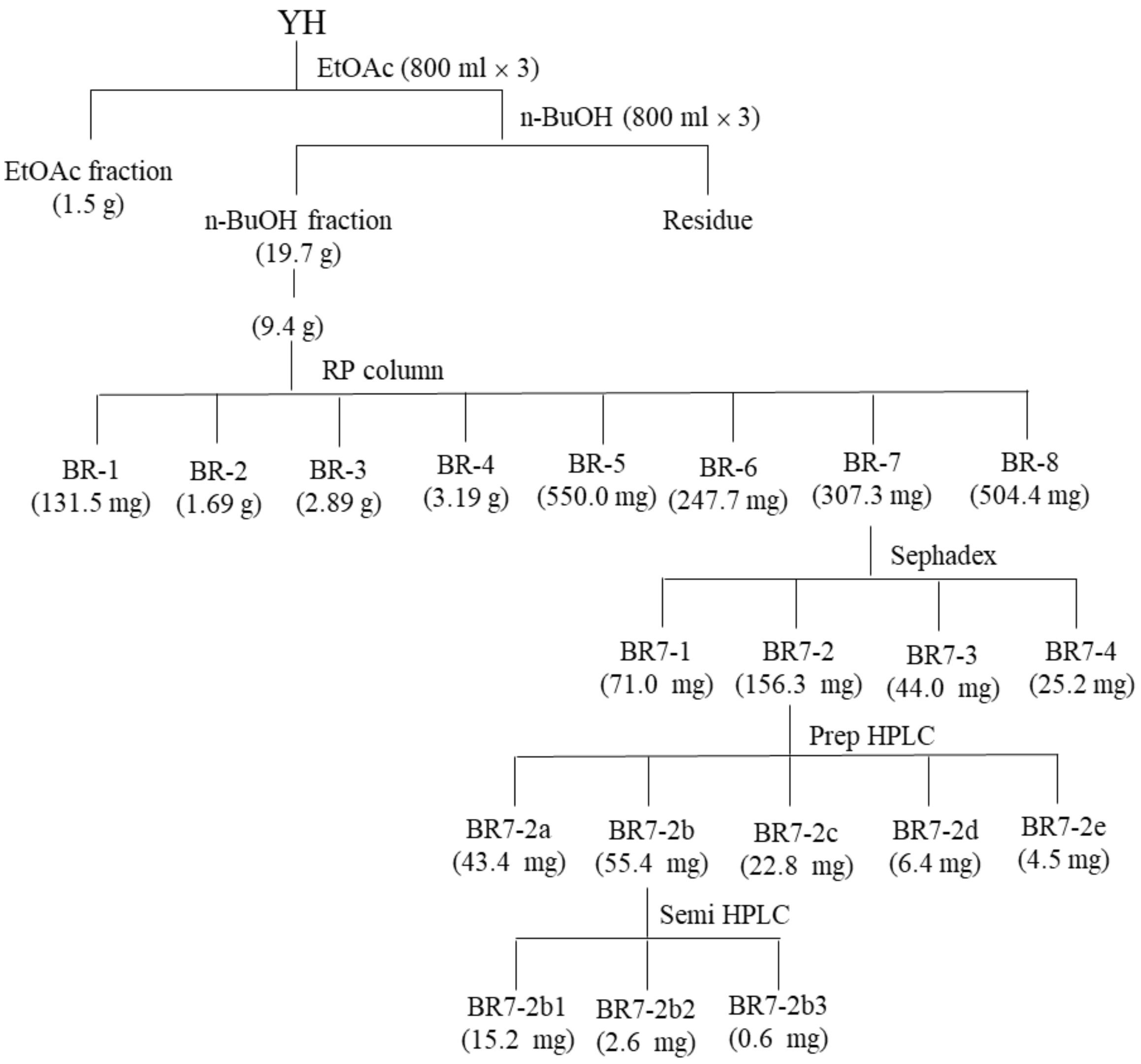

Figure S1. Isolation of anti-adipogenic compounds from yeast hydrolysate (YH).

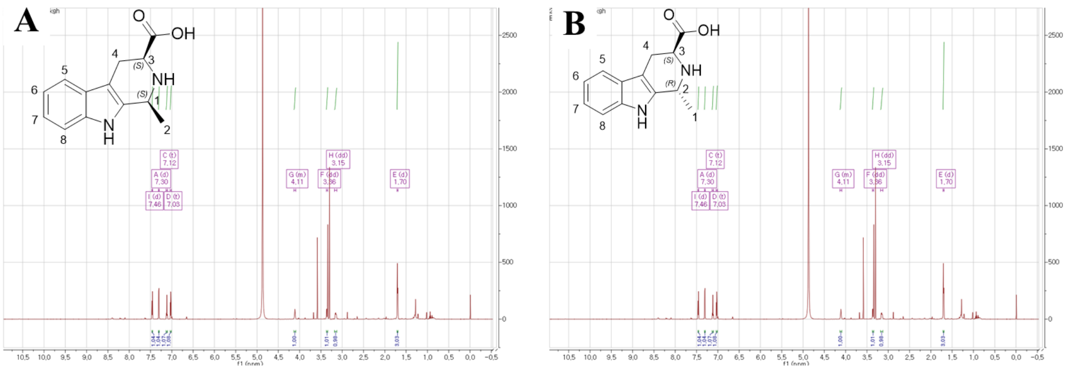

Figure S2. 1H-NMR spectrum of BR7-2b1 (a) and BR7-2b2 (b).

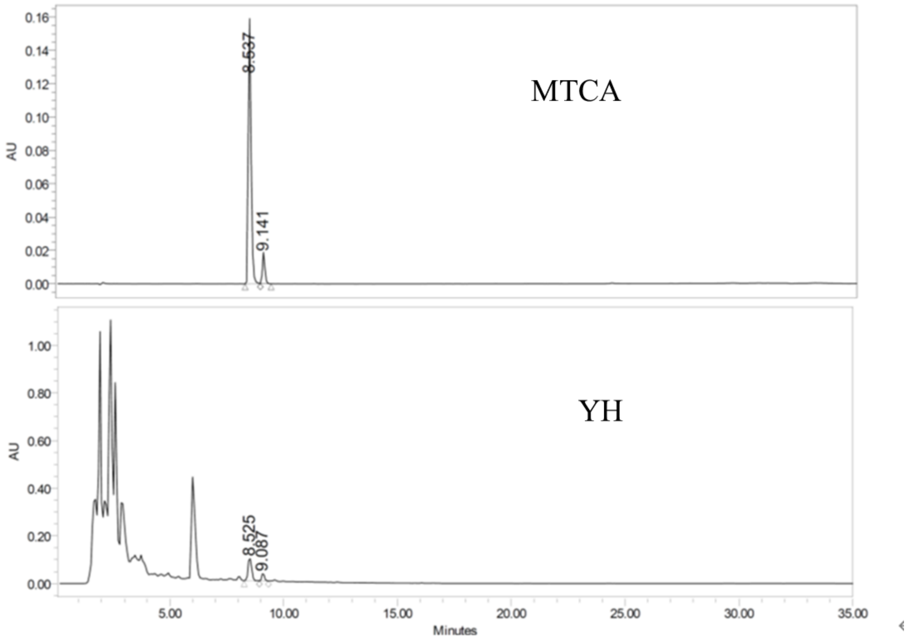

Figure S3. Chromatograms of 1-methyl-1,2,3,4-tetrahydro-β-carboline-3-carboxylic acid (MTCA) standard (a) and YH (b).
